# Passive Leg-Raising Test as a Predictor for the Drop in Blood Pressure After a Lumbar Epidural Block in the Pain Clinic: A Prospective Observational Study

**DOI:** 10.3390/jcm14082629

**Published:** 2025-04-11

**Authors:** Cho-Long Kim, Seung-Wan Hong, Yun-Do Jung, Sung-Yeon Jung, Seong-Hyop Kim

**Affiliations:** 1 Department of Anesthesiology and Pain Medicine, Hanyang University Medical Center, Hanyang University School of Medicine, 222, Wangsimni-ro, Seongdong-gu, Seoul 04763, Republic of Korea; starkcl@naver.com (C.-L.K.); fcfrankfurty@gmail.com (Y.-D.J.); jungsy0519@gmail.com (S.-Y.J.); 2 Department of Anesthesiology and Pain Medicine, Konkuk University Medical Center, Konkuk University School of Medicine, 120-1 Neungdong-ro, Hwayang-dong, Gwangjin-gu, Seoul 05030, Republic of Korea; 20150077@kuh.ac.kr; 3 Department of Infection and Immunology, Konkuk University School of Medicine, Seoul 05029, Republic of Korea; 4 Department of Medicine, Institute of Biomedical Science and Technology, Konkuk University School of Medicine, Seoul 05029, Republic of Korea; 5 Department of Medical Education, Konkuk University School of Medicine, Seoul 05029, Republic of Korea

**Keywords:** passive leg-raising test, blood pressure, epidural block, hypotension, low back pain, adverse effects

## Abstract

**Background:** Hypotension following lumbar epidural blocks is a concern in pain management, and predicting this complication remains challenging. **Methods:** Patients who received a transforaminal lumbar epidural block were enrolled. Before the epidural block, the PLRT was performed, and systolic, diastolic, and mean BP were measured. The BP measurements were taken again after the epidural block. The correlations between changes in BP before and after the PLRT, and changes in BP before and after the epidural block, were analyzed. The risk factors for a greater than 20% decrease in the mean BP after the epidural block were also analyzed. **Results:** The changes in BP, except diastolic BP before and after the PLRT, were correlated with the changes in the BP before and after the epidural block. Patients with more than a 20% decrease in mean BP after the epidural block had significantly lower height, underlying hypertension, and a higher mean change in BP during the PLRT than the patients without. The optimal threshold values for height and change in the mean BP during the PLRT to predict more than a 20% decrease in the mean BP after the epidural block, based on the receiver operating characteristic curve analysis, were 156.5 cm for height and 5.5 mmHg for the change in mean BP during the PLRT. **Conclusions:** The PLRT before a lumbar epidural block was useful in predicting the decrease in BP after a lumbar epidural block.

## 1. Introduction

An epidural block is one of the most common procedures in the pain clinic to alleviate back pain, sciatica, or leg pain [1,2,3,4,5,6]. However, an epidural block with a local anesthetic agent is associated with unwanted side effects. The decrease in blood pressure (BP) after an epidural block is the most common clinical manifestation of epidural block-induced sympathetic blockade. It frequently occurs at the site of the epidural block, although it is dependent on the patient’s characteristics, the level of the block, and other factors [7,8,9]. The decrease in BP after an epidural block does not cause a problem, except minor discomfort, but it can lead to hypotension and related symptoms, such as dizziness, nausea, and vomiting. Severe hypotension can result in loss of consciousness and hemodynamic collapse. Despite the circumstances, a tool to predict the degree of the drop in BP after an epidural block has not been developed.

Passive leg raising (PLR) is a well-known exercise to manage shock, as it increases venous return from the leg by gravity, which increases cardiac output, according to Starling’s law [10], with a higher BP. Therefore, the PLR test (PLRT) has been commonly utilized to assess fluid responsiveness [11]. The volume of venous return is dependent on the venous reservoir in the legs. Epidural block-induced sympathetic blockade decreases venous return from the legs [12]. It is the main cause of the drop in BP after an epidural block. Therefore, the PLRT performed before an epidural block may be useful to predict the drop in BP after an epidural block. Although venous pooling in the legs after an epidural block is one of the main causes of the drop in BP after an epidural block, no study has investigated the association between the change in BP using PLR before the epidural block and the change in BP after an epidural block.

We hypothesized that performing the PLRT before an epidural block would predict the drop in BP after a lumbar epidural block. This study was designed to evaluate the association between the change in BP using the PLRT before a lumbar epidural block and the change in BP after a lumbar epidural block. We also evaluated the change in BP using the PLRT before the lumbar epidural block, to predict the drop in BP after the epidural block.

## 2. Materials and Methods

### 2.1. Study Population

This study is a prospective observational study conducted during January to November 2023. After obtaining approval from the Institutional Review Board (The Institutional Review Board of Hanyang University Medical Center, Seoul, Republic of Korea; Reference No, 2022-11-040-002; Date of Approval, 27 December 2022) and informed consent from the patients, this study was registered at the Clinical Research Information Service, Korea Centers for Disease Control and Prevention, Ministry of Health and Welfare (KCT0008097; date of registration, 9 January 2023; http://cris.nih.go.kr (accessed on 7 April 2025)) following the principles of the 2013 Declaration of Helsinki.

Patients who received a lumbar transforaminal epidural block in the pain clinic were enrolled in this study. The inclusion criteria were as follows: (1) adults aged 20–80 years, (2) back pain or radiating leg pain, and (3) the decision to undergo lumbar transforaminal epidural block as a part of clinical management. Exclusion criteria were: (1) patients aged under 20 or over 80, (2) allergy to the contrast agent used during the procedure, (3) pain during the physical examination process in the outpatient setting, specifically when the straight leg raise test (SLRT) elicits pain at an angle of 45 degrees or less, and (4) refusal to participate in the study or inability to provide informed consent.

### 2.2. Exposure and Measurements

Upon arrival in the operating room, the patient was laid on a folding bed in the supine position at 0°. BP monitoring and pulse oximetry (Masimo Corp., Irvine, CA, USA) were established. BP was measured at baseline (T_0_), and the PLRT was performed after the measurement [11]. Both legs were raised at 45° to return venous blood to the heart for 30 s, and BP was measured at PLR (T_PLRT_).

The exposure in this study is the BP change during the PLRT, which is the key factor we are investigating to predict the subsequent BP change after the lumbar epidural block. After the PLRT and BP measurements, the patient’s position was changed to the 0° prone position for the epidural block. After the epidural block, the patient was repositioned to the 0° supine position. The patient was transferred to the recovery room in a mobile bed and closely observed for 30 min. BP was measured 30 min after the epidural block (T1). The time point was chosen because several studies have reported that the lowest BP reduction typically occurs between 10 to 30 min post-procedure. Since observation in the recovery room takes place after the procedure and discharge typically occurs after 30 min, BP was measured at the 30-min time point, just before discharge [13,14]. After confirming the absence of any epidural block-related complications, the patient was discharged.

The outcome of this study is the BP change after the epidural block, which is the primary variable we are examining in relation to the changes observed during the PLRT. The study was designed based on the assumption that BP is related to fluid status, and considering this, BP changes before and after PLRT would be similar to those before and after a lumbar epidural block.

Pain was checked at T_0_ and T_1_ using a visual analog pain scale. The scale was presented as a 10 cm line ranging from 0 (no pain) to 100 (the worst pain imaginable). Hypotension-related symptoms, such as dizziness, nausea, vomiting, and sweating, were checked during the stay in the recovery room.

### 2.3. Transforaminal Lumbar Epidural Block

The procedures were scheduled in the morning, and the patients arrived 30 min early to ensure a stable state in the waiting room before entering the operating room. The procedure was performed under fluoroscopic guidance in an operating room. The patient was placed in the prone position. A pillow was placed under the abdomen. The target level and number for the epidural block were determined by history taking and a physical examination with radiological findings. Using a fluoroscope (Cios^®^ Select, SIEMENS Healthineers, Forchheim, Germany), anteroposterior, lateral, and oblique views of the spine were obtained to determine and verify the entry point. After sterile skin preparation and local infiltration with 2% lidocaine (1 mL at the entry point), a 23-gauge Quincke needle was inserted for the epidural block and advanced under fluoroscopic guidance. Once the needle was positioned, the epidural space was confirmed by injecting OMNIPAQUE 300™ (iohexol, GE Healthcare, Milwaukee, WI, USA) as a contrast agent, using real-time fluoroscopy. There was no spread of the contrast agent into the intrathecal or intravascular space. Following confirmation of the epidural space, 5 mL of 0.2% ropivacaine (Rocaine Inj™, Reyon Pharmaceutical Co., Seoul, Republic of Korea), 2.5 mg of dexamethasone (Dexamethasone Inj™, Yuhan, Seoul, Republic of Korea), and 500 international units of hyaluronidase (Hirax Inj™, BMI Korea, Seoul, Republic of Korea) were slowly administered into the epidural space for each block, with great care. After the medication was administered, the needle was removed, and the patient was transferred to the recovery room in a mobile bed.

### 2.4. Statistical Analysis

The primary outcome was the correlation between the change in mean BP before and after the PLRT (T_0_ and T_PLRT_), and the change in mean BP before and after the epidural block (T_0_ and T_1_). The change in the mean BPs between T_0_ and T_PLRT_ in a pilot study with 10 patients who received a transforaminal lumbar epidural block for back pain (86 ± 1 mmHg at T_0_ and 93 ± 4 mmHg at T_PLRT_) was 7 ± 4 mmHg, and the change in the mean BP between T_0_ and T_1_ was (86 ± 1 mmHg at T_0_ and 71 ± 2 mmHg at T_1_) was 15 ± 2 mmHg. The sample size of 34 for the primary outcome was calculated to achieve a power of 0.9 and an α-value of 0.05, using G-Power 3.1.9.7 (Universität Kiel, Kiel, Germany) [15,16].

Statistical analyses were performed using IBM SPSS version 29.0 (Statistical Package for Social Sciences, Chicago, IL, USA). First, the demographic data were analyzed according to the >20% drop in mean BP after the epidural block. Categorical variables were analyzed using the chi-square or Fisher’s exact test, and continuous variables were analyzed using the independent *t*-test or the Mann–Whitney test, depending on the normality test. Second, univariate analysis for each potential risk factor for more than a 20% drop in mean BP after an epidural block was performed using logistic regression. Factors with *p* < 0.05 in the univariate analysis were subjected to a multivariate conditional logistic regression model using the backward stepwise regression procedure. Odds ratios (ORs) with 95% confidence intervals (CIs) were calculated based on the final model. Third, the cutoff values for the changes in mean BP, to distinguish responders (>20% drop in mean BP) vs. non-responders, were obtained after the epidural block. The area under the curve (AUC) was determined by receiver operating characteristic (ROC) curve analysis, calculated based on the multivariable model.

Data are expressed as the number of patients, mean ± standard deviation, or median [interquartile range]. A *p*-value < 0.05 was considered significant.

## 3. Results

**Figure 1:**
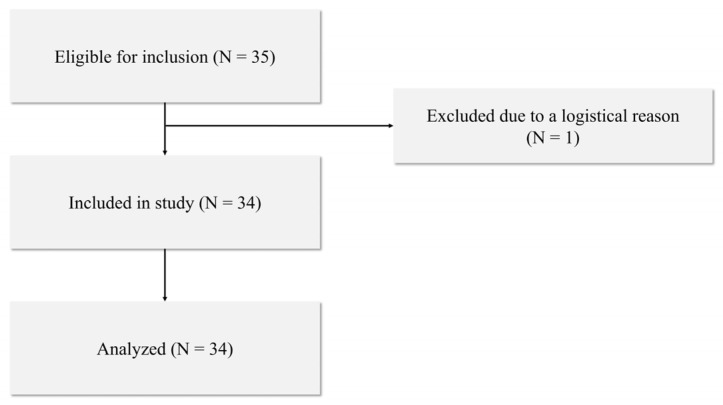
A flow diagram of patient recruitment and selection.

In total, 34 patients were enrolled in the study, but one was excluded from the final analysis due to a technical problem (loss of signed informed consent form) (Figure 1). Demographic data of the 34 patients are summarized in Table 1.

**Figure 2:**
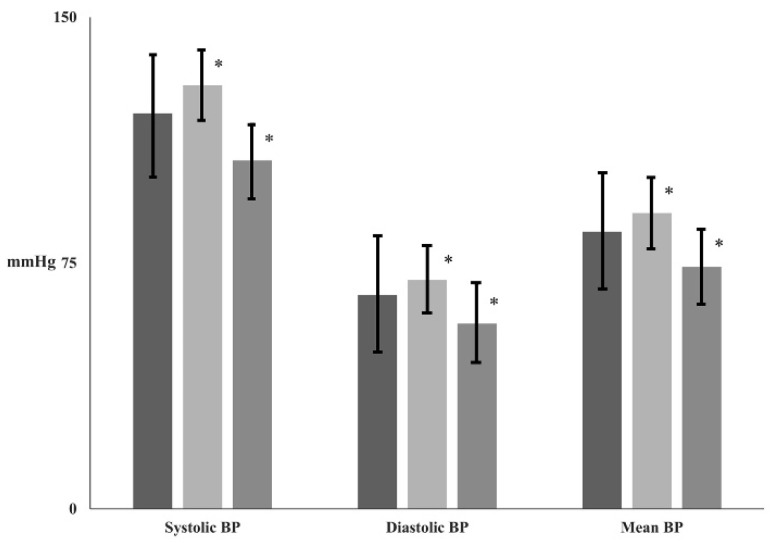
The changes of all blood pressures (BPs) before and after epidural block. Abbreviation: T_0_ (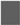), baseline; T_PLRT_ (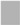), at passive leg-raising test; T_1_ (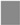), at 30 min after epidural block. *: *p* < 0.001 compared with T_0_.

PLR increased all BPs (the BPs at T_PLRT_), compared with the BPs at T_0_ (systolic BP, 120 ± 18 mmHg → 129 ± 18 mmHg, *p* < 0.001; diastolic BP, 65 ± 11 mmHg → 70 ± 10 mmHg, *p* < 0.001; mean BP, 85 ± 11 mmHg → 90 ± 12 mmHg, *p* < 0.001) (Figure 2) (Table 2).

After the epidural block, all BPs (the BPs at T_1_) decreased, compared with the BPs at T_0_ (systolic BP, 120 ± 18 mmHg → 106 ± 18 mmHg, *p* < 0.001; diastolic BP, 65 ± 11 mmHg → 57 ± 11 mmHg, *p* < 0.001; mean BP, 85 ± 11 mmHg → 74 ± 12 mmHg, *p* < 0.001) (Figure 2).

The patients with more than a 20% drop in mean BP after the epidural block were significantly older and smaller than the patients without the BP drop (Table 3). They had a significantly larger change in the mean BP between T_0_ and T_PLRT_ (Table 3). They also had significantly greater changes in all BPs between T_0_ and T_1_ (Table 3).

**Figure 3:**
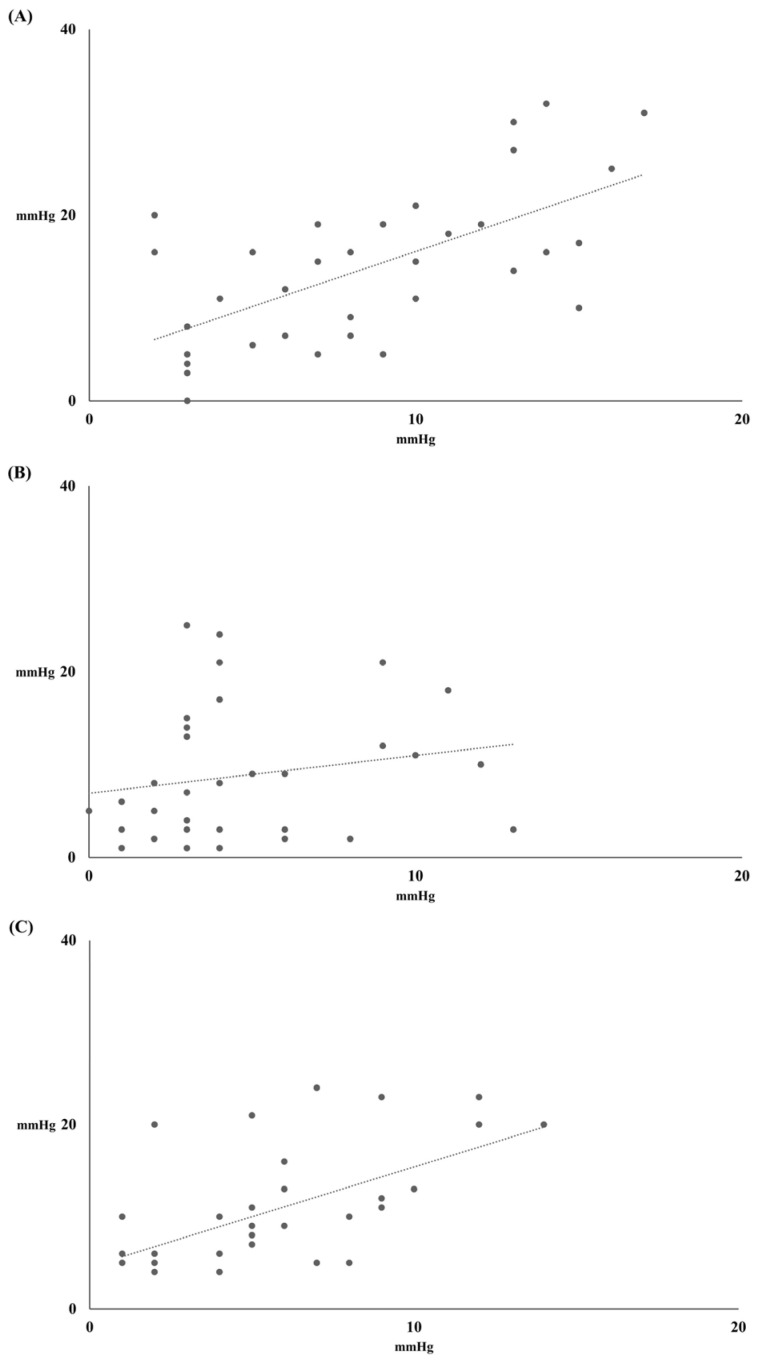
Correlation between change of all blood pressures (BPs) before and after passive leg-raising test (PLRT) (*X*-axis) and change of all BPs before and after epidural block (*Y*-axis). (**A**) Systolic BP (y = 1.19X + 4.24, r = 0.638, *p* < 0.001), (**B**) Diastolic BP (y = 0.41X + 6.92, r = 0.196, *p* = 0.266) and (**C**) Mean BP (y = 1.08X + 4.60, r = 0.568, *p* < 0.001).

The changes in all BPs, except diastolic BP between T_0_ and T_PLRT_, were correlated with the changes in all BPs except diastolic BP between T_0_ and T_1_ (systolic BP, r = 0.638, *p* < 0.001; diastolic BP, r = 0.196, *p* = 0.266; mean BP, r = 0.568, *p* < 0.001) (Figure 3).

Univariate analysis identified height, hypertension as an underlying disease, and the change in mean BP between T_0_ and T_PLRT_ as risk factors for a > 20% drop in mean BP after the epidural block. Multiple logistic regression analysis also identified height [OR 0.783 (95% CI, 0.614–0.998), *p* = 0.048], hypertension as an underlying disease [OR 24.560 (95% CI, 1.131–533.504), *p* = 0.042], and change in mean BP between T_0_ and T_PLRT_ [OR 1.653 (95% CI, 1.058–2.581, *p* = 0.027] as risk factors for more than a 20% drop after the epidural block (Table 4).

**Figure 4:**
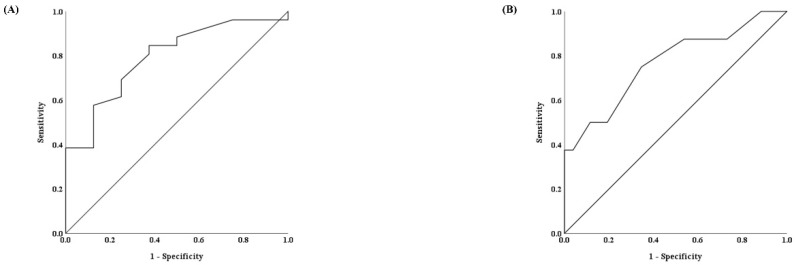
Receiver operating characteristic curves for height (**A**) and change of mean blood pressure (BP) before and after passive leg-raising test (PLRT) (**B**).

The AUC, calculated from the multivariable model using the results presented in Table 4, for predicting the mean BP drop after the lumbar epidural block, was 0.793 (95% CI, 0.624–0.962; *p* = 0.013) and 0.767 (95% CI, 0.564–0.970; *p* = 0.024), respectively (Figure 4).

The optimal threshold values for height and the change in mean BP between T_0_ and T_PLRT_, to predict more than a 20% drop in mean BP after the epidural block based on the ROC curve analysis were 156.5 cm (sensitivity, 62.5%, and specificity, 84.6%) for height and 5.5 mmHg (sensitivity, 75.0%, and specificity, 65.4%) for the change in mean BP between T_0_ and T_PLRT_ (Figure 4).

## 4. Discussion

The present study showed that changes in all BPs, except diastolic BP before and after the PLRT, were correlated with changes in all BPs before and after the epidural block. The risk factors for more than a 20% drop in mean BP after the epidural block were height, hypertension as an underlying disease, and the change in mean BP between T_0_ and T_PLRT_.

An epidural block is commonly performed in a pain clinic. Although the procedure is safe, the decrease in BP after the procedure should be monitored [17]. If the lower BP after the procedure cannot be managed, it may lead to a safety accident, such as a fall-down, although a lower BP is usually transient and a case with additional management is rare. The patient demographics of pain clinics show a high proportion of elderly patients, many of whom have coexisting degenerative aortic stenosis. In these patients, a decrease in systemic vascular resistance (SVR) due to epidural block can lead to catastrophic outcomes. Therefore, utilizing methods to predict hypotension, as demonstrated in this study, is clinically important. However, no study has tried to predict the decrease in BP after an epidural block in the pain clinic, as only studies on conventional neuraxial blocks for surgical procedures have been published [18,19]. Moreover, those studies only reported qualitative results, not quantitative results. Previous studies have shown that high-level blockade (≥T5) and old age (≥40 years) are the two main factors in hypotensive complications after spinal anesthesia, with an incidence of 15.3% to 33% [19,20].

Several methods are used to assess fluid responsiveness. Static indices like central venous pressure (CVP) and pulmonary artery occlusion pressure (PAOP) have limitations, while dynamic indices such as stroke volume variation (SVV), pulse pressure variation (PPV), and respiratory changes in inferior vena cava diameter are more accurate, especially in mechanically ventilated patients. Echocardiography is a useful non-invasive tool for predicting fluid responsiveness. Echocardiographic methods to predict fluid responsiveness include the assessment of inferior vena cava (IVC) collapsibility, superior vena cava (SCV) collapsibility, and respiratory variations in aortic blood flow velocity [21]. Previous studies have reported that prespinal ultrasound scanning of the inferior vena cava collapsibility index (IVCCI) is a reliable predictor of hypotension following spinal anesthesia, with a cutoff point of >42% [22]. Another study found that IVCCI was correlated with the amount of fluid administered (r^2^ = 0.32) [23]. In pain clinics, ultrasound-guided procedures are widely performed alongside C-arm-guided procedures. Since ultrasound-based IVC assessment has been reported as an important parameter for evaluating blood volume status, it could be considered an alternative to PLRT in this study.

PLRT is effective in assessing fluid responsiveness in both spontaneously breathing and mechanically ventilated patients [21]. Increased venous return, using PLR, is “auto-transfusion”. Considering that the lower BP after the epidural block is not actual hypovolemia but functional hypovolemia due to the decreased sympathetic tone [24], the PLRT is thought to be an easy and practical tool to predict the drop in BP after an epidural block.

In the present study, the risk factors for more than a 20% drop in mean BP after the epidural block were height, hypertension as an underlying disease, and change in mean BP between T_0_ and T_PLRT_.

We used a fixed dose of ropivacaine for the epidural block in all enrolled patients. Therefore, it was reasonable that the fall in BP was negatively correlated with the height of the patient, as demonstrated in previous studies [25,26].

High BP is associated with structural and functional changes in the vascular system and the heart. Hypertension increases the production of collagen fiber and accelerates the degradation of elastin fiber in the vascular system [27]. These changes increase the thickening of the wall in the artery, the remodeling of arteries with a higher ratio of wall to lumen, and decreased microcirculation. These changes in the vascular system lead to increased afterload. Hypertension also increases the workload of the heart, inducing structural and functional changes in the myocardium. The changes in the myocardium lead to left ventricular hypertrophy with diastolic dysfunction, leading to a stiff heart. Finally, stroke volume increases in patients with hypertension. Therefore, patients with hypertension are susceptible to a lower BP after sympathetic blockade [28,29]. This finding indicates that hypertension, one of the risk factors for more than a 20% drop in mean BP after an epidural block in the present study, was reasonable.

Epidural block is usually administered via one of two approaches, such as the interlaminar approach or the transforaminal approach. The transforaminal approach has been clinically performed for selective blocks but is associated with a larger drop in BP [9]. In the present study, only the transforaminal approach was used. However, the decrease in BP was not severe, but was not compared according to the approach. The decrease could have been associated with the amount or the concentration of the drugs used for the epidural block.

The threshold values of 156.5 cm for height and 5.5 mmHg for BP change are clinically significant. A height of 156.5 cm was linked to a higher risk of a greater than 20% BP drop after the epidural block. Additionally, a 5.5 mmHg BP change during PLRT was a reliable predictor of significant hypotension, helping clinicians anticipate and manage BP fluctuations to improve patient safety. Since the AUC for height was greater than the AUC for the change in mean BP between T_0_ and T_PLRT_ in the present study, using PLRT alone may not be the most effective method to predict a more than 20% drop in BP. However, combining the simple and intuitive PLRT test with height information may provide useful insights into BP changes after the epidural block.

This study has several limitations. (1) This study is the relatively small sample size (*n* = 34), which may have contributed to the wide confidence interval for hypertension as a risk factor. A larger sample size could potentially lead to a more precise estimate and narrower confidence intervals, thus improving the reliability of the results. (2) In this study, a fixed drug dose was used regardless of the individual characteristics of the patients. As in previous studies, the fall in BP was negatively correlated with the height of the patient. Considering this characteristic, if different drug doses had been used for each patient, more definitive results might have been obtained in predicting the extent of blood pressure reduction after epidural block using the PLRT. (3) According to the usage guidelines for Ropivacaine, a standard dose for pain control was used. Considering the number of segments blocked per mL and the fact that the procedure was performed using a selective transforaminal approach at a specific level, the expected block level can be estimated. However, investigating the exact blocked levels would have improved the quality of the study.

The PLRT is a bedside assessment to determine fluid responsiveness. Fluid responsiveness is defined as an increase in the stroke volume after fluid loading. The definite assessment of fluid responsiveness is a fluid challenge. Therefore, fluid loading is recommended to prevent the drop in BP before or after an epidural block.

In conclusion, the PLRT before the epidural block was a useful tool for predicting the drop in BP after a lumbar epidural block in the pain clinic. More than a 20% drop in mean BP after the lumbar epidural block was associated with low patient height, hypertension as an underlying disease, and a higher mean BP change during the PLRT.

## Figures and Tables

**Table 1:** Demographic data.

Category	Variable		
Personal Information			
	Gender (F/M)		20 (59%)/14 (41%)
	Age (years)		63 [55–71]
	Height (cm)		162 ± 10
	Weight (kg)		60 [53–73]
Underlying dz			
	None		17 (50%)
	HTN		12 (35%)
	IHD		3 (9%)
	DM		3 (9%)
	Dyslipidemia		3 (9%)
Diagnosis			
	HNP		18 (53%)
	Spinal stenosis		12 (35%)
	FBSS		2 (6%)
	Others		2 (6%)
Current medication			
	None		9 (26%)
	ARB		8 (24%)
	BB		6 (18%)
	CCB		9 (26%)
	Antiplatelet		9 (26%)
	Dyslipidemia medication		3 (9%)
	DM medication		2 (6%)
	Gabapentinoid		13 (38%)
	NSAID		11 (32%)
	Analgesic except NSAID		3 (9%)
	Muscle relaxant		7 (21%)
	TCA		9 (26%)
Epidural block			
	Side		
		Left	15 (44%)
		Right	11 (32%)
		Both	8 (24%)
	Level		
		L4	3 (9%)
		L5	10 (29%)
		S1	2 (6%)
		L4 & L5	4 (12%)
		L5 & S1	13 (38%)
		L4, L5 & S1	2 (6%)
	Number		
		1	9 (26%)
		2	21(62%)
		3	3 (9%)
		4	1 (3%)
VAS (0–100)			
	Before epidural block		60 ± 19
	After epidural block		5 [0–30]
Change of BP			
	between T_0_ and T_PLRT_		
		Systolic	9 ± 4
		Diastolic	4 [3–6]
		Mean	6 ± 3
	between T_0_ and T_1_		
		Systolic	14 ± 8
		Diastolic	7 [3–13]
		Mean	9 [6–14]
Hypotension-related Sx			
	Dizziness		3 (9%)
	Nausea		0 (0%)
	Vomiting		0 (0%)
	Sweating		0 (0%)

Data is expressed as number of patients, median [interquartile range] or mean ± standard deviation. The percentages in the categories of underlying diseases and current medications may exceed 100% because patients may have multiple underlying diseases or be taking multiple medications simultaneously. **Abbreviations:** F, female; M, male; dz, disease; HTN, hypertension; IHD, ischemic heart disease; DM, diabetes mellitus; HNP, herniated nucleus pulposus; FBSS, failed back surgery syndrome; NSAID, non-steroidal anti-inflammatory drug; TCA, tricyclic anti-depressant; VAS, visual analogue pain scale; BP, blood pressure; T_0_, baseline; TPLRT, at passive leg-raising test; T_1_, at 30 min after epidural block; Sx, symptom.

**Table 2:** Blood pressure during the procedure.

	T_0_ (Baseline)	T_PLRT_ (at Passive Leg-Raising Test)	T_1_ (at 30 min After Epidural Block)
Systolic BP (mmHg)	120 ± 18	129 ± 18	106 ± 18
Diastolic BP (mmHg)	65 ± 11	70 ± 10	57 ± 11
Mean BP (mmHg)	85 ± 11	90 ± 12	74 ± 12

**Table 3:** Demographic data according to the existence of above 20% drop of mean blood pressure (BP) after lumbar epidural block.

			Mean BP Drop O	Mean BP Drop X	*p* Value
Gender (F/M)			7/1	13/13	0.102
Age (years)			71 [62–79]	61 [37–69]	0.035
Height (cm)			155 ± 7	164 ± 10	0.017
Weight (kg)			54 [52–69]	60 [54–80]	0.177
Underlying dz					
	None		2	15	0.249
	HTN		6	6	0.013
	IHD		1	2	1.000
	DM		0	3	1.000
	Dyslipidemia		2	1	0.131
Diagnosis					
	HNP		3	15	0.429
	Spinal stenosis		4	8	0.410
	FBSS		1	1	0.421
	Others		0	2	1.000
Current medication					
	None		0	9	0.077
	ARB		4	4	0.066
	BB		3	3	0.126
	CCB		4	5	0.165
	Antiplatelet		2	7	1.000
	Dyslipidemia		2	1	0.131
	DM		0	2	1.000
	Gabapentinoid		4	9	0.69
	NSAID		1	10	0.227
	Analgesic except NSAID		0	3	1.000
	Muscle relaxant		2	5	1.000
	TCA		1	8	0.403
Epidural block					
	Side				1.000
		Left	4	11	
		Right	2	9	
		Both	2	6	
	Level				0.767
		L4	1	2	
		L5	3	7	
		S1	1	1	
		L4 & L5	0	4	
		L5 & S1	3	10	
		L4, L5 & S1	0	2	
	Number				0.242
		1	4	5	
		2	3	18	
		3	1	2	
		4	0	1	
VAS (0–100)					
	Before epidural block		68 ± 23	58 ± 17	0.196
	After epidural block		10 [0–28]	5 [0]	1.000
Change of BP between T_0_ and T_PLRT_					
	Systolic		11 ± 5	8 ± 4	0.094
	Diastolic		4 [3–10]	3 [2–6]	0.110
	Mean		8 ± 4	5 ± 3	0.007
Change of BP between T_0_ and T_1_					
	Systolic		24 ± 7	11 ± 6	<0.001
	Diastolic		20 [16–23]	5 [3–8]	<0.001
	Mean		21 [20–23]	7 [5–10]	<0.001
Hypotension-related Sx					
	Dizziness		1	2	1.000
	Nausea		0	0	-
	Vomiting		0	0	-
	Sweating		0	0	-

Data is expressed as number of patients, mean ± standard deviation or median [interquartile range]. **Abbreviations:** F, female; M, male; dz, disease; HTN, hypertension; IHD, ischemic heart disease; DM, diabetes mellitus; HNP, herniated nucleus pulposus; FBSS, failed back surgery syndrome; NSAID, non-steroidal anti-inflammatory drug; TCA, tricyclic anti-depressant; VAS, visual analogue pain scale; BP, blood pressure; T_0_, baseline; T_PLRT_, at passive leg-raising test; T_1_, at 30 min after epidural block; Sx, symptom.

**Table 4:** Predictors for 20% drop of mean blood pressure (BP) after epidural block.

	Variables	Univariate Analysis		Multivariate Analysis	
		Odd Ratio (95% CI)	*p* Value	Odd Ratio (95% CI)	*p* Value
Gender (F/M)		7.000 (0.751–65.221)	0.087		
Age (years)		1.090 (0.994–1.194)	0.067		
Height (cm)		0.874 (0.773–0.989)	0.032	0.783 (0.614–0.998)	0.048
Weight (kg)		0.961 (0.898–1.027)	0.237		
Underlying disease					
	HTN	10.000 (1.585–63.097)	0.014	24.560 (1.131–533.504)	0.042
	IHD	1.714 (0.135–21.820)	0.678		
	DM	0 (0–0)	0.999		
	Dyslipidemia	8.333 (0.644–107.851)	0.105		
Diagnosis					
	HNP	0.440 (0.086–2.244)	0.323		
	Spinal stenosis	2.250 (0.447–11.334)	0.326		
	Failed back surgery syndrome	3.571 (0.197–64.632)	0.389		
	Others	0 (0–0)	0.999		
Current medication					
	ARB	5.500 (0.958–31.589)	0.056		
	BB	4.600 (0.709–29.841)	0.110		
	CCB	4.200 (0.771–22.869)	0.097		
	Antiplatelet	0.905 (0.147–5.583)	0.914		
	Antihyperlipidemic Drugs	8.333 (0.644–107.85)	0.105		
	DM medication	0 (0–0)	0.999		
	Gabapentinoid	1.889 (0.380–9.395)	0.437		
	NSAIDs	0.229 (0.024–2.146)	0.196		
	Analgesic except NSAID	0 (0–0)	0.999		
	Muscle Relaxants	1.400 (0.215–9.121)	0.725		
	TCA	0.321 (0.034–3.064)	0.324		
Injection side					
	Left	6.559 (0.242–177.823)	0.229		
	Right	1.364 (0.278–6.683)	0.702		
	Both	1.111 (0.176–7.011)	0.911		
Injection level					
	L4	1.714 (0.135–21.820)	0.678		
	L5	1.629 (0.306–8.679)	0.568		
	S1	3.571 (0.197–64.632)	0.389		
	L4 & L5	0 (0–0)	0.999		
	L5 &S1	0.960 (0.187–4.924)	0.961		
	L4, L5 & S1	0 (0–0)	0.999		
Injection Number		0.425 (0.107–1.692)	0.225		
The number of injection levels		0.369 (0.081–1.678)	0.197		
Injection agents					
	Ropivacaine	0.918 (0.800–1.054)	0.225		
	Dexamethasone	0.710 (0.409–1.234)	0.225		
	Hyaluronidase	0.998 (0.996–1.001)	0.225		
Change of BP between T_0_ and T_PLRT_			0.725		
	Systolic	1.178 (0.968–1.434)	0.103		
	Diastolic	1.171 (0.931–1.471)	0.177		
	Mean	1.426 (1.058–1.922)	0.020	1.653 (1.058–2.581)	0.027

**Abbreviations:** F, female; M, male; dz, disease; HTN, hypertension; IHD, ischemic heart disease; DM, diabetes mellitus; HNP, herniated nucleus pulposus; FBSS, failed back surgery syndrome; NSAID, non-steroidal anti-inflammatory drug; TCA, tricyclic anti-depressant; VAS, visual analogue pain scale; BP, blood pressure; T_0_, baseline; T_PLRT_, at passive leg-raising test; T_1_, at 30 min after epidural block; Sx, symptom.

## Data Availability

The data that support the findings of this study are available on request from the corresponding author. The data are not publicly available due to privacy or ethical restrictions.
